# Mortality Risk of Colistin vs. Non-Colistin Use in Cancer Patients with Multidrug-Resistant Gram-Negative Bacterial Infections: Stratified by Resistance Profile and Concomitant Medications

**DOI:** 10.3390/medicina61081361

**Published:** 2025-07-28

**Authors:** Soo Hyeon Lee, Yongwon Choi, Chang-Young Choi, Yeo Jin Choi, Sooyoung Shin

**Affiliations:** 1 Department of Regulatory Science, Graduate School, Kyung Hee University, Seoul 02447, Republic of Korea; 2 Institute of Regulatory Innovation through Science (IRIS), Kyung Hee University, Seoul 02447, Republic of Korea; 3 Department of Hematology-Oncology, School of Medicine, Ajou University, Suwon 16499, Republic of Korea; dreamliebe@ajou.ac.kr; 4 Department of Gastroenterology, Ajou University Hospital, Suwon 16499, Republic of Korea; 5 Department of Pharmacy, College of Pharmacy and Institute of Integrated Pharmaceutical Sciences, Kyung Hee University, Seoul 02447, Republic of Korea; 6 Department of Pharmacy, College of Pharmacy, Ajou University, Suwon 16499, Republic of Korea; 7 Research Institute of Pharmaceutical Science and Technology (RIPST), Ajou University, Suwon 16499, Republic of Korea

**Keywords:** colistin, cancer, mortality, multi-drug resistant, Gram-negative bacteria, immunocompromised

## Abstract

*Background and Objectives:* Cancer patients are particularly susceptible to infections caused by multidrug-resistant Gram-negative bacteria (MDR GNB) due to chemotherapy- or radiation therapy-induced immunosuppression. Colistin is often prescribed as a last-resort agent for MDR GNB infection, but its clinical benefit in oncology patients remains unclear. This study aims to evaluate the mortality risk associated with colistin versus non-colistin regimens in cancer patient with MDR GNB infections, stratified by resistance profiles, infection sites, and concomitant medication use. *Materials and Methods:* A retrospective cohort study was conducted in adult cancer patients with MDR GNB infections that are resistant to at least three antibiotic classes and identified from at least two anatomical sites at a tertiary care hospital in Korea. Propensity score-matched in a 1:3 ratio either to the colistin group or non-colistin group and multivariate Cox hazard regression analyses were used to evaluate mortality in cancer patients with MDR GNB infections, primarily *Acinetobacter baumannii, Klebsiella pneumoniae,* and *Pseudomonas aeruginosa*. *Results:* A total of 85 patients (29 patients in the colistin and 56 patients in the non-colistin group) were included in the analysis. Overall, colistin use did not show a statistically significant mortality benefit compared to non-colistin regimens (hazard ratio (HR) 0.93, 95% CI 0.47–1.87). However, the subgroup analysis revealed that colistin had a potential association with significantly lower mortality in pneumonia patients with aminoglycoside-resistant infections (HR 0.04, 95% CI 0.002–0.69). Concomitant use of antipsychotics and benzodiazepines in selected resistance profiles also correlated with improved outcomes. In contrast, a potential association was found between concomitant macrolide use and increased mortality in patients with fluoroquinolone- or penicillin-resistant profiles. *Conclusions:* Colistin may offer survival benefits in selected high-risk cancer patients with MDR GNB pneumonia. Treatment outcomes are influenced by resistance profiles, infection sites, and concomitant medications, indicating the significant importance of individualized antimicrobial therapy and antimicrobial stewardship in oncology patients.

## 1. Introduction

Antibiotic resistance (AR) has emerged as a critical global public health threat, contributing to an estimated 4.71 million deaths from antimicrobial-resistant infections in 2021 [1]. The incidence of AR has increased significantly since the COVID-19 pandemic, with multidrug-resistant (MDR) strains accounting for approximately 60.8% of co-infections among COVID-19 patients [2]. Among various MDR organisms, Gram-negative bacteria (GNB) are particularly concerning due to their distinctive outer membrane structures, intrinsic resistance mechanisms, and the limited availability of effective antibiotic treatments [3]. Notably, MDR GNB organisms, including *Acinetobacter baumannii, Klebsiella pneumoniae,* and *Pseudomonas aeruginosa*, are frequently associated with healthcare-acquired infections [4,5]. These organisms often produce extended-spectrum β-lactamases (ESBLs) and carbapenemases, which induce high levels of resistance against multiple antibiotic classes, including carbapenems and β-lactam/β-lactamases inhibitor combinations [1,5]. Their ability to survive in a healthcare environment poses a substantial threat to patient safety and global public health [1,4,6].

Cancer patients are particularly vulnerable to infections, including those caused by MDR GNB organisms, due to their immunocompromised status, frequent hospitalizations, and intensive exposure to broad-spectrum antibiotics [7,8]. Both chemotherapy and radiation therapy can induce neutropenia and disrupt mucosal barriers; moreover, the use of invasive devices such as central venous catheters (CVCs) further increases the risk of bloodstream and respiratory tract infections [8,9]. Despite substantial healthcare burdens associated with MDR GNB infections, including high morbidity and mortality rates, prolonged hospital stays, and significantly increased healthcare costs, antibiotic treatment options remain limited, and clinical outcomes in these patients are often poor [3,10,11]. Moreover, considering diverse chemotherapy regimens administered to cancer patients, significant alterations in pharmacokinetic (PK) and pharmacodynamic (PD) profiles may occur, potentially impacting the efficacy and safety of antibiotic therapy [12,13,14]. Furthermore, drug–drug interactions, organ dysfunction, and altered drug metabolism can complicate antibiotic treatment, posing additional challenges in treating MDR GNB infections in this population [14,15].

Colistin (polymyxin E) is frequently prescribed to manage carbapenem-resistant *Pseudomonas aeruginosa* (CRPA) and carbapenem-resistant *Acinetobacter baumannii* (CRAB) [11]. However, its clinical use is often complicated by nephrotoxicity and PK variability [3]. A recent meta-analysis demonstrated that although a clinical cure was associated with lower mortality, serum colistin concentrations at steady state did not differ significantly between clinically cured and non-cured patients, nor between those who did and did not develop nephrotoxicity [3]. Notably, patients with respiratory tract infections, which are commonly observed in cancer patients, exhibited markedly lower clinical cure rates compared to those with urinary tract infections [16]. These findings imply the therapeutic complexity of colistin, emphasizing the need for rigorous evaluation of its clinical outcomes across diverse patient populations and anatomical sites of infection. Therefore, this study aims to investigate the mortality risk associated with colistin versus non-colistin antibiotic regimens in cancer patients with multisite MDR GNB infections.

## 2. Materials and Methods

### 2.1. Study Design and Study Population

This was a retrospective cohort study utilizing electronic medical records (EMR) of Ajou University Medical Center, a tertiary care hospital in Korea, in accordance with Strengthening the Reporting of Observational Studies in Epidemiology (STROBE) guideline [17]. Adult cancer patients (aged 18 years or older) with a documented history of MDR bacterial infection, as confirmed by antibiotic susceptibility testing, were included. Antibiotic susceptibility test was performed using a combination of methods—broth microdilution (BMD), E-test, and the VITEK 2 system—depending on institutional availability and time period. Patients who were diagnosed with cancer or received chemotherapy between January 2010 and December 2022 were eligible for inclusion. Cancer diagnoses were identified by the International Classification of Diseases, Tenth Revision (ICD-10) codes, with codes C00-C97 considered for analysis. Patients with at least two positive cultures from different anatomical sites, including the genitourinary system, respiratory system, and blood samples obtained on at least two separate dates, were included. Prespecified exclusion criteria included patients younger than 18 years, those with a history of dialysis, pregnant or lactating women, and individuals who did not receive chemotherapy following surgery. Cases with antibiotic susceptibility test results indicating ‘resistant’ or ‘intermediate’ were excluded from the analysis. Additionally, cultures obtained from anatomical sites other than blood, sterile body fluids, and genitourinary and respiratory specimens were excluded from the analysis. The study protocol was reviewed and approved by the Institutional Review Board (IRB) of Ajou University Hospital (AJOUIRB-DB-2023-219, approved 9 May 2023), given the retrospective design and use of de-identified patient data. The requirement for informed consent and additional ethical approval was waived by the IRB.

### 2.2. Study Cohort and Study Outcomes

MDR GNB infection was defined as any infection caused by *Acinetobacter baumannii*, *Klebsiella pneumoniae*, or *Pseudomonas aeruginosa*, with MDR classified as resistance to at least three classes of antibiotics [18]. The results of antibiotic susceptibility tests were reviewed for all patients in the analysis, and only cases in which the administered antibiotic was classified as ‘susceptible’ at the minimum inhibitory concentration (MIC) level were considered clinically appropriate and included in the cohort. Patients who received colistin for the treatment of the identified infection were included in the colistin cohort, while those who did not receive colistin were classified into the non-colistin cohort. To ensure temporal relevance between bacterial resistance and treatment, only patients who were administered antibiotics within 30 days following the first antibiotic susceptibility test with MDR GNB isolation were included in the cohort. Importantly, only patients who were prescribed and administered antibiotics in accordance with clinical guidelines and approved product information, in terms of dose and treatment duration, were included in the analysis. Cases with incomplete or incorrect administration were excluded. All exposure classification and statistical analyses were based on actual administered antibiotic data, as verified through the institution’s EMR system. The primary outcome was mortality during the follow-up period, and antibiotic resistance type, antibiotic types, cancer type, and concomitant medication use were separately captured along with patient mortality over the study period as secondary outcomes. The first day of antibiotic treatment was assigned as the index date, and patient follow-up began on the index date until the earliest of the following: study outcome, follow-up discontinuation, or end of the study period (31 December 2022). To balance potential confounding between colistin and non-colistin users, eligible patients were matched using propensity score (PS) matching at a 1:3 ratio to the colistin and non-colistin cohorts, based on relevant pretreatment attributes in terms of age, sex, antibiotic susceptibility testing results, concomitant medications including antibiotics, cancer types, and comorbidities.

### 2.3. Covariates

Prespecified covariates included age, sex, antibiotic class, cancer type, comorbidities, and concomitant medications. Cancer diagnosis and comorbidities were defined using ICD-10 codes. Comorbidities included cardiovascular disease (CVD), diabetes, hypertension, liver disease, renal failure, pneumonia, and cerebrovascular disease. Concomitant medications included antibiotics, nonsteroidal anti-inflammatory drugs (NSAIDs), opioids, hypnotics/sedatives, immunosuppressants, antidepressants, antipsychotics, anesthetics, anxiolytics, and benzodiazepines.

### 2.4. Statistical Analysis

Patients were divided into the colistin and non-colistin cohorts based on the first antibiotic use after isolation of MDR GNB in antibiotic susceptibility testing. PSs for individual patients were estimated using a logistic regression model that included the following pre-specified covariates: age, sex, cancer type, comorbidities, and concomitant medications, including benzodiazepines, penicillin, carbapenems, glycopeptides, and oxazolidinones. PS matching was performed at a 1:3 ratio using nearest neighbor matching with a caliper of 0.2, and balance between the colistin and non-colistin groups was assessed using standardized mean differences (SMDs) with a threshold of <0.1. Fisher’s exact test was performed to assess differences in baseline characteristics between the colistin and non-colistin cohorts, and *p*-values were reported accordingly. Kaplan–Meier survival curves and log-rank tests were utilized to compare mortality between the two cohorts. Survival analysis was performed using Cox proportional hazards models. Variables included in the multivariate model were selected based on assessments for correlation and multicollinearity; variables with a variance inflation factor (VIF) ≥ 10 were excluded to minimize redundancy and instability in the model. The time-to-event was defined as the number of days from the index date to death and was analyzed in 28-day intervals, corresponding approximately to monthly periods. The PS-matched study participants were further stratified by baseline attributes reflecting patient factors or disease severity, such as concomitant drug types, cancer types, comorbidities, and antibiotic susceptibility test results. Subgroup analysis was performed at 168 days (approximately 6 months) post-index date to explore early mortality trends. All statistical tests with *p*-values < 0.05 were considered statistically significant. Analyses were conducted using R (version 4.4.0) and SPSS Statistics (version 29.0.2; IBM SPSS Statistics for Windows, Armonk, NY, USA).

## 3. Results

### 3.1. Characteristics of Study Patients

**Figure 1:**
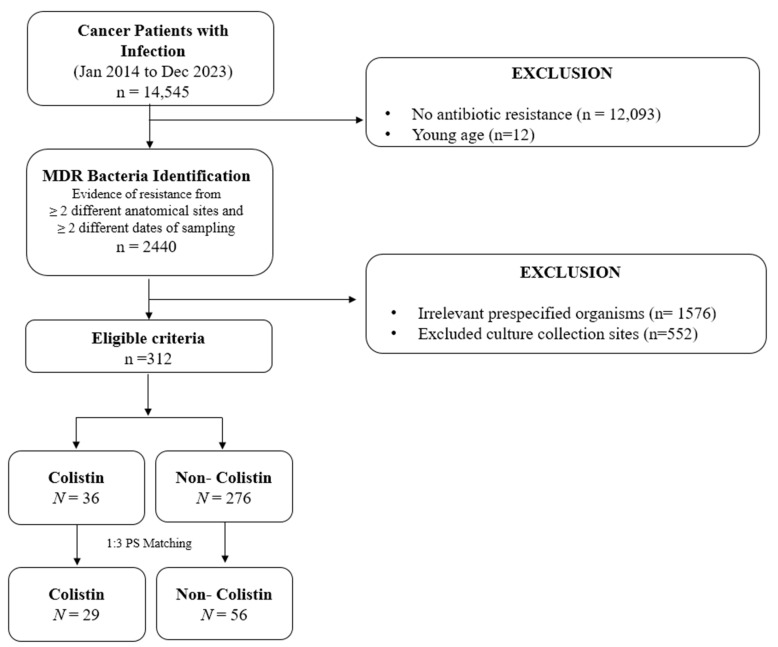
Flow chart of the selected study patients.

A total of 14,545 cancer patients who received chemotherapy underwent antibiotic susceptibility tests between 2014 and 2023 (Figure 1). Among them, patients with MDR bacterial isolates identified in at least two antibiotic susceptibility tests, each obtained from different anatomical sites and collected on separate dates, were included in the initial cohort, resulting in 2440 patients. After filtering for eligible microorganisms and appropriate culture collection sites, 312 patients were identified, including 36 colistin users and 276 non-colistin users. Following 1:3 PS matching, 85 patients, 29 patients in the colistin and 56 patients in the non-colistin group, were finally selected for the study analyses. The baseline characteristics of included patients are summarized in Table 1. No significant differences in baseline characteristics were observed between groups after PS matching, with respect to age, sex, culture sites, isolated microorganisms, antibiotic resistance profiles, cancer types, comorbidities, and concomitant medication types. None of the patients in the matched cohort had diagnoses of renal cell carcinoma or malignancies involving the nervous system. Additionally, only two patients in the non-colistin group had non-malignant renal or urinary tract conditions. Baseline characteristics prior to PS matching and SMD before and after PS matching are presented in Tables S1 and S2.

### 3.2. Study Outcomes

**Figure 2:**
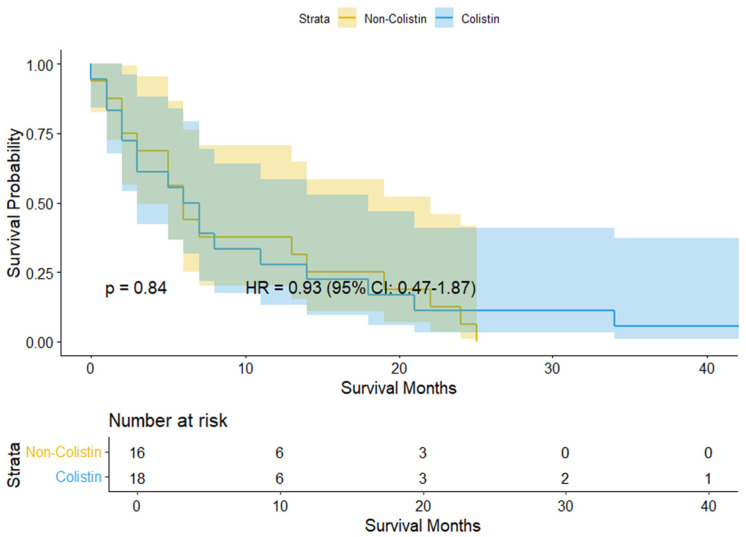
Kaplan–Meier curve of mortality in cancer patients with MDR GNB infections.

The Kaplan–Meier curve for cancer patients with MDR GNB infection is presented in Figure 2. No significant difference in mortality risk was observed between the colistin and non-colistin groups (HR 0.93, 95% CI 0.47–1.87).

The multivariate Cox analysis revealed no significant differences in the risk of mortality between the colistin and non-colistin groups across major comorbidities, including cardiovascular disease, diabetes mellitus, liver disease, and nephropathy (Table 2). Notably, even among patients with sepsis or septic shock, mortality risk remained comparable between the groups.

**Figure 3:**
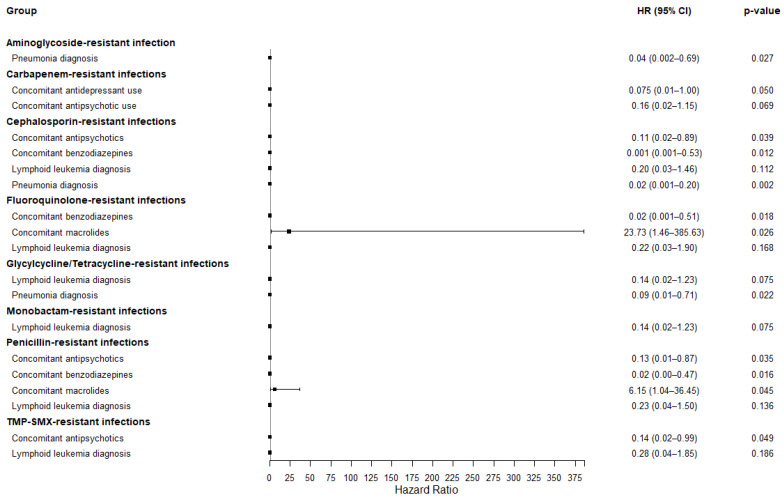
PS-matched analysis for mortality risk associated with colistin vs. non-colistin use, stratified by antibiotic resistance profile.

Several covariates were significantly associated with mortality risk in the PS-matched analysis stratified by antibiotic resistance profiles (Figure 3). Among patients with aminoglycoside- or glycycline-resistant infection, colistin use was associated with a substantially reduced mortality risk in those with pneumonia (HR 0.04, 95% CI 0.002–0.69). In patients with cephalosporin-resistant infections, the colistin group demonstrated substantially lower mortality risk in those receiving concomitant antipsychotics (HR 0.11, 95% CI 0.02–0.89) or benzodiazepines (HR 0.001, 95% CI 0.001–0.53), as well as in patients with pneumonia (HR 0.02, 95% CI 0.001–0.20). Among patients with fluoroquinolone-resistant infection, colistin use was associated with a significantly lower mortality risk in those receiving benzodiazepines (HR 0.02, 95% CI 0.001–0.51), whereas mortality risk was substantially increased with concomitant use of macrolides (HR 23.73, 95% CI 1.46–385.63). A similar trend was observed in patients with penicillin-resistant infection: colistin use was linked to a higher mortality risk with macrolides (HR 6.15, 95% CI 1.04–36.45), but a lower risk with concomitant use of antipsychotics (HR 0.13, 95% CI 0.01–0.87) or benzodiazepines (HR 0.02, 95% CI 0.00–0.47).

### 3.3. Subgroup Analysis on 168-Day (6-Month) Mortality

Risk factors associated with elevated 168-day (6-month) mortality are summarized in Table 3. Multivariate Cox regression analysis identified aminoglycoside-resistant infection (HR 54.50, 95% CI 3.30–900.20) and concomitant penicillin use (HR 51.91, 95% CI 2.83–950.60) as significant predictors of increased mortality in cancer patients with MDR GNB infections.

**Figure 4:**
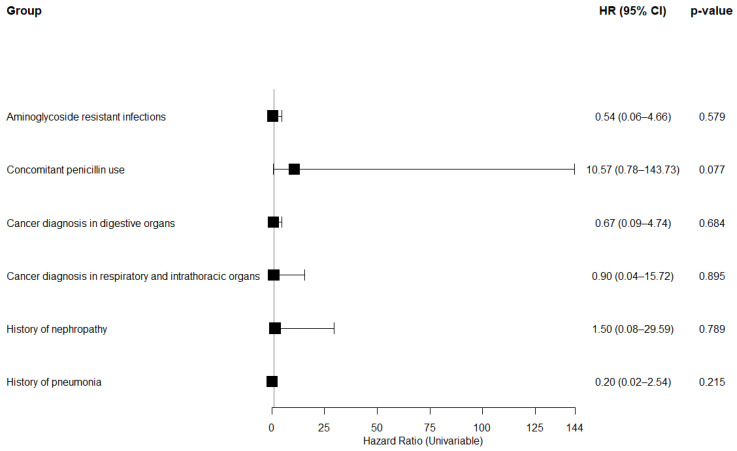
PS-matched analysis for 168-day (6-month) mortality risk associated with colistin vs. non-colistin use in cancer patients.

However, no significant differences in mortality risk between the colistin and non-colistin groups were observed across the following covariates: aminoglycoside-resistant infections, concomitant penicillin use, cancer involving digestive organs or respiratory and intrathoracic organs, and a history of nephropathy or pneumonia (Figure 4).

## 4. Discussion

This study evaluated the mortality risk associated with colistin vs. non-colistin antibiotic regimens in cancer patients with MDR GNB infections involving at least two anatomical sites. While no significant difference in mortality was observed between the colistin and non-colistin groups, stratified analyses revealed important subgroup-specific associations. Notably, among patients with aminoglycoside- or glycycline-resistant infections, colistin use was associated with a substantially lower mortality risk in those with respiratory disease, primarily pneumonia. Similarly, in cephalosporin-resistant infections, colistin conferred a substantial survival benefit in patients receiving concomitant antipsychotics or benzodiazepines, as well as in those with pneumonia infection. In contrast, this study revealed a potential association between colistin use and increased mortality among patients receiving concomitant macrolides, particularly in those with either fluoroquinolone- or penicillin-resistant infections. Multivariate analysis of 168-day mortality further identified aminoglycoside resistance and concomitant penicillin use as strong predictors of poor prognosis. However, no significant differences in mortality were observed between colistin and non-colistin treatments across covariates, including resistance type, primary cancer sites, and comorbidities.

As colistin is often reserved for the treatment of MDR infections, evidence on its clinical outcomes, particularly in vulnerable populations such as cancer patients undergoing chemotherapy, remains limited and inconclusive. Several studies have reported comparable clinical efficacy between colistin and either beta-lactam antibiotics or quinolones in the treatment of MDR *Pseudomonas aeruginosa* [19,20]. Moreover, a recent prospective cohort study demonstrated no significant differences in mortality or infection recurrence rates between treatment groups; however, patients treated with tigecycline experienced shorter hospital stays and treatment durations compared to those with carbapenem-resistant *Enterobacteriaceae* (CRE) infections treated with colistin. These findings suggest that while colistin remains an important therapeutic option in the management of antimicrobial resistance, its comparative clinical effectiveness versus alternative antibiotics likely depends on patient-specific characteristics, anatomical sites of infection, and the resistance profiles of the causative microorganisms.

According to our previous pharmacovigilance study on drug-induced fatalities, antibiotic-induced adverse drug reactions (ADRs) were among the most frequently reported causes of fatal outcomes, particularly in vulnerable populations such as older adults [21]. Moreover, aging, antibiotic class, and concomitant medications were identified as major predictors for increased fatality risk [21]. These findings emphasize the importance of cautious antibiotic prescribing, particularly in high-risk patients. In the present study, although colistin did not demonstrate an overall mortality benefit compared to non-colistin regimens, its impact varied significantly depending on the infection site, resistance phenotype, and concomitant medication use. The observed survival benefit in pneumonia patients with aminoglycoside-resistant infections suggests that colistin may serve as a valuable therapeutic option in selected high-risk patients where alternative antibiotic agents are limited (Figure 2). Nonetheless, clinical decision-making should be guided by a stratified approach that considers resistance profile, anatomical sites and types of concomitant medications, including other concomitant antibiotics, to minimize potential risk and enhance clinical prognosis.

Interestingly, a possible association between concomitant use of macrolides and increased mortality was observed in patients with fluoroquinolone- or penicillin-resistant infections (Figure 2). However, the finding should be interpreted with caution due to wide confidence intervals, which may reflect limited sample size or heterogeneity within the subgroup. While macrolides have some activity against GNB, these agents are not typically recommended as the first choice for treating MDR GNB strains [22]. Macrolide use in these cases may reflect empirical or adjunctive antibiotic prescribing practices rather than targeted therapy [23]. Cancer patients are frequently exposed to febrile neutropenia and are particularly susceptible to infections caused by atypical pathogens, such as *Haemophilus influenzae*, *Mycoplasma pneumoniae*, *Moraxella catarrhalis*, and *Neisseria* species [16,24,25]. As macrolides provide coverage against atypical or co-infecting organisms, macrolides may have been administered to patients with suspected polymicrobial or atypical infections as part of empiric therapy, emphasizing the importance of appropriate empiric antibiotic selection to optimize clinical outcomes [26]. Furthermore, the rising incidence of macrolide-resistant organisms may further complicate treatment decisions and contribute to suboptimal responses or adverse effects in immunocompromised patients [27]. These concerns also indicate the potential adverse drug interactions, altered PK/PD characteristics, or confounding by indication in patients with severe medical conditions. While the association between macrolide use and increased mortality is intriguing, the clinical plausibility remains uncertain and may be the result of residual confounding factors such as disease severity, indication bias, or underlying functional status. Therefore, cautious evaluation of empirical antibiotic combinations and implementation of appropriate antimicrobial stewardship are warranted, particularly in vulnerable populations. Meanwhile, further investigations are required to clarify the causal relationship between macrolide use and increased mortality risk in resistant infections, considering potential confounding factors such as disease severity and co-infections. Additionally, future studies should evaluate the clinical effectiveness of macrolides across different cancer subtypes and resistance profiles within oncology populations.

The use of antipsychotics has been associated with an increased risk of respiratory infections, such as pneumonia, particularly in patients with schizophrenia [28,29]. Although the precise mechanism of antipsychotic-induced respiratory infection remains unclear, several factors may contribute to increased risk of respiratory infections, including acute dystonia from dopamine receptor blockage, respiratory collapse, sedation-induced hypoventilation, and anticholinergic-mediated reduction in mucociliary clearance [30,31]. Moreover, antipsychotic use in medically complex patients, such as those with cancer or an immunocompromised condition, may exacerbate infection risk due to overlapping vulnerabilities. Notably, antipsychotics are frequently prescribed beyond their approved indications, especially in hospitalized or palliative care settings, where they are often used off-label to manage agitation, delirium, or sleep disturbances [30,32]. Interestingly, in our study, colistin-treated patients who were concomitantly administered antipsychotics had some potential association with lower mortality risk, particularly in cases involving cephalosporin- or penicillin-resistant infection (Figure 2). This unexpected finding may reflect unmeasured confounding, disease severity, underlying functional status, differences in clinical management or supportive care, or potential immunomodulatory or neuroprotective effects of certain psychotropic agents. Hence, further research is warranted to clarify the role of antipsychotics in infection outcomes, particularly in high-risk patients such as older adults and immunocompromised patients.

Interestingly, this study revealed a potential association between concomitant benzodiazepine use and a lower mortality risk with colistin among cancer patients, particularly in those with cephalosporin- or penicillin-resistant infections (Figure 2). However, this finding should be interpreted with caution due to the small sample size and clinically implausible hazard ratios. Benzodiazepines are also frequently prescribed off-label to manage anxiety, agitation, and insomnia [33], and their use is particularly common in hospitalized or medically complex patients. However, the accumulating evidence suggests that incident benzodiazepine use is associated with an increased risk of serious infections [34], and chronic use may further elevate the risk of infection-related hospitalization, including COVID-19 [34,35]. Several pharmacological effects of benzodiazepine, including sedation-induced hypoventilation, impaired mucociliary clearance, and suppressed immune function, may compromise host defense mechanisms against respiratory and systemic infections [36]. These risks may be amplified in cancer patients and other immunocompromised individuals who are already highly susceptible to infectious complications [37]. A recent clinical study indeed demonstrated reduced survival of pancreatic cancer patients exposed to lorazepam, implying that benzodiazepine exposure may negatively affect the clinical prognosis of oncology populations. While a potential protective association was observed in this study, the clinical plausibility of this effect still needs to be determined. Considering the attenuated immune responses and frequent polypharmacy in cancer patients, the observed reduction in mortality in this study may reflect confounding by indication, differences in supportive care practices, or unrecognized pharmacodynamic interactions. Hence, further research is warranted to clarify the role of benzodiazepines in infection-related outcomes and to determine whether these findings are consistent across cancer types and levels of disease severity. Meanwhile, judicious prescribing of benzodiazepines is strongly recommended in high-risk patients to minimize potential infection-related harm.

Lastly, the multivariate analysis revealed that aminoglycoside-resistance and concomitant penicillin use were independent predictors of increased 168-day mortality, regardless of colistin use. Preliminary analyses showed no significant differences in outcomes within the 30-day window, although the underlying reason remains unclear. This finding aligns with prior data indicating that short-term mortality in immunocompromised cancer patients with sepsis or septic shock is substantial, estimated around 50–60% at 30 days, but continues to rise over time, often exceeding 80% by one year [38]. Therefore, we adopted a 168-day follow-up period to better capture delayed clinical trajectory and long-term outcomes in this high-risk population. These findings suggest that resistance phenotypes and the cumulative antimicrobial burden may play a more critical role in determining long-term outcomes than the specific use of colistin or alternative agents. These results highlight the importance of individualized treatment strategies that incorporate both microbiological profiles and patient-specific clinical factors to guide optimal antimicrobial treatment in high-risk populations. Hence, further research is needed to validate these predictors in larger, controlled clinical trials and to investigate the mechanistic pathways by which antimicrobial resistance and polypharmacy influence long-term survival in cancer patients with MDR infections.

This study has several limitations that should be acknowledged. First, the findings may not be generalized to similar patient populations in other institutions, as this was a retrospective observational study from a single institution. In addition, the inclusion criteria, which were limited to cancer patients with severe or complicated infections caused by GNB resistant to at least three classes of antibiotics from at least two anatomical sites, enabled the recruitment of a high-risk population but also contributed to a relatively small sample size (85 patients). The limited small sample size may have reduced the statistical power of subgroup analyses and increased the risk of overfitting. As a result, some hazard ratios yielded extreme values with wide confidence intervals, indicating limitations in model robustness. Moreover, multiple subgroup analyses were conducted without correction for multiple comparisons due to the limited sample size. These subgroup findings should therefore be interpreted as exploratory. Additionally, while interaction effects between specific concomitant medications such as macrolides, benzodiazepines, or antipsychotics, and resistance phenotypes on mortality were observed, these associations may be confounded by factors such as underlying disease severity, indication bias, or functional status, all of which are difficult to fully control in a retrospective dataset. The clinical plausibility of these findings remains uncertain, and prospective validation in larger cohorts is needed to determine whether these drug-resistance interactions represent true clinical risk modifiers or reflect residual confounding. Nonetheless, the strength of this study was that it used rigorous PS matching to minimize baseline differences between treatment groups, thereby enhancing the internal validity of findings considering the heterogeneity of the cancer patient population. Another important limitation of this study is the potential under-detection of colistin heteroresistance due to the antibiotic susceptibility testing methods. Although we utilized a combination of antibiotic susceptibility testing methods that are commonly used in clinical practice, these methods have known limitations in detecting heteroresistant subpopulations, particularly in *Acinetobacter baumannii* and *Klebsiella pneumoniae.* Misclassification of heteroresistant isolates as susceptible could have led to ineffective treatment in some patients receiving colistin, potentially biasing the comparison of outcomes between treatment groups. Hence, further study evaluating colistin efficacy should incorporate standard and highly sensitive methods to accurately detect heteroresistance and minimize misclassification bias. Moreover, missing, incomplete, or inaccurately entered information may have introduced information bias, potentially affecting the reliability of clinical information. As with all observational studies, the possibility of residual confounding cannot be excluded, including unmeasured clinical severity, treatment intent, and the timing of antibiotic initiation. Furthermore, we were not able to assess cause-specific mortality due to inconsistent documentation. However, among the 36 recorded mortalities, only four patients (three in the non-colistin group and one in the colistin group) had documented diagnoses of terminal malignancy, which may likely be the primary cause of death in these cases. Despite these limitations, this study has several notable strengths. It is among the few to evaluate the clinical impact of colistin versus non-colistin regimens on mortality in cancer patients with MDR GNB infections, incorporating a range of resistance phenotypes and stratified analyses by infection site and concomitant medication use. The use of PS-matched analysis and multivariate models enhanced the robustness of the finding by accounting for potential confounding variables. Additionally, the use of real-world data from a heterogeneous cancer population supports the potential generalizability of the findings to clinical practice.

## 5. Conclusions

This study revealed the complexity of managing MDR GNB infections in cancer patients, who are vulnerable to frequent infection due to chemotherapy- or radiation therapy-induced immunosuppression. While no overall mortality benefit was observed with colistin use compared to non-colistin antibiotic regimens, stratified analyses revealed potential clinical advantages of colistin in specific high-risk subgroups, particularly among aminoglycoside-resistant pneumonia cases. Conversely, concomitant macrolide administration was associated with increased mortality, emphasizing the importance of selecting appropriate antibiotics based on both resistance profiles and concomitant medications. These findings reinforce the importance of antimicrobial stewardship in oncology care. Nonetheless, further controlled studies are warranted to validate the current findings to support evidence-based treatment strategies for MDR infections in immunocompromised populations.

## Figures and Tables

**Table 1:** Baseline demographic characteristics of the patients.

Characteristics	No. of Cases (% Relative Frequency) or Median (IQR)			
	Total (n = 85)	Non-Colistin (n = 56)	Colistin (n = 29)	*p*-Values
**Age (years)**	65.49 ± 11.49	65.34 ± 12.23	64.64 ± 12.81	>0.05
30~39	3 (3.53%)	1 (1.79%)	2 (6.90%)	
40~49	7 (8.24%)	5 (8.93%)	2 (6.90%)	
50~59	14 (16.47%)	9 (16.07%)	5 (17.24%)	
60~69	24 (28.24%)	16 (28.57%)	8 (27.59%)	
70~79	30 (35.29%)	21 (37.50%)	9 (31.03%)	
80~89	7 (8.24%)	4 (7.14%)	3 (10.34%)	
**Sex**				
Men	62 (72.94%)	40 (71.43%)	22 (75.86%)	>0.05
Women	23 (27.06%)	16 (28.57%)	7 (24.14%)	
**Culture**				
Blood/Fluid	12 (14.12%)	8 (14.29%)	4 (13.79%)	>0.05
Genital/Urinary	8 (9.41%)	8 (14.29%)	0 (0.00%)	
Respiratory	36 (42.35%)	25 (44.64%)	11 (37.93%)	
Duplicate	29 (34.12%)	15 (26.79%)	14 (48.28%)	
**Microorganism**				
*Acinetobacter baumannii*	23 (27.06%)	13 (23.21%)	10 (34.48%)	>0.05
*Klebsiella pneumoniae*	20 (23.53%)	15 (26.79%)	5 (17.24%)	
*Pseudomonas aeruginosa*	15 (17.65%)	10 (17.86%)	5 (17.24%)	
≥2 cultures	27 (31.76%)	18 (32.14%)	9 (31.03%)	
**Antibiotic resistance**				
Aminoglycoside	61 (10.93%)	40 (10.96%)	21 (10.88%)	>0.05
Carbapenems	65 (11.65%)	42 (11.51%)	23 (11.92%)	
Cephalosporin	71 (12.72%)	46 (12.60%)	25 (12.95%)	
Colistin	2 (0.36%)	1 (0.27%)	1 (0.52%)	
Fluoroquinolone	73 (13.08%)	47 (12.88%)	26 (13.47%)	
Glycylcycline	48 (8.60%)	32 (8.77%)	16 (8.29%)	
Monobactam	64 (11.47%)	42 (11.51%)	22 (11.40%)	
Penicillin	76 (13.62%)	51 (13.97%)	25 (12.95%)	
Tetracycline	18 (3.23%)	11 (3.01%)	7 (3.63%)	
TMP_SMX	80 (14.34%)	53 (14.52%)	27 (13.99%)	
**Concomitant antibiotics**				
Aminoglycoside	44 (5.48%)	30 (5.78%)	14 (4.93%)	>0.05
Carbapenem	68 (8.47%)	43 (8.29%)	25 (8.80%)	
Cephalosporin	78 (9.71%)	51 (9.83%)	27 (9.51%)	
Fluoroquinolones	57 (7.10%)	38 (7.32%)	19 (6.69%)	
Glycopeptide	76 (9.46%)	49 (9.44%)	27 (9.51%)	
Lincosamides	13 (1.62%)	8 (1.54%)	5 (9.51%)	
Macrolide	17 (2.12%)	11 (2.12%)	6 (0.70%)	
Monobactam	1 (0.12%)	1 (0.19%)	0 (0.00%)	
Oxazolidinone	8 (1.00%)	6 (1.16%)	2 (0.70%)	
Penicillin	73 (9.09%)	47 (9.06%)	26 (9.15%)	
Rifamycin	8 (1.00%)	5 (0.96%)	3 (1.06%)	
Tetracycline	6 (0.75%)	2 (0.39%)	4 (1.41%)	
TMP_SMX	11 (1.37%)	5 (0.96%)	6 (2.11%)	
**Comedications**				
Anesthetics	64 (7.97%)	42 (8.09%)	22 (7.75%)	>0.05
Antidepressants	20 (2.49%)	15 (2.89%)	5 (1.76%)	
Antipsychotic	50 (6.23%)	32 (6.17%)	18 (6.34%)	
Anxiolytics	6 (0.75%)	3 (0.58%)	3 (1.06%)	
Benzodiazepine	71 (8.84%)	46 (8.86%)	25 (8.80%)	
Buprenorphine	1 (0.12%)	1 (0.19%)	0 (0.00%)	
Hypnotics and sedatives	18 (2.24%)	11 (2.12%)	7 (2.46%)	
Immunosuppressants	8 (1.00%)	3 (0.58%)	5 (1.76%)	
NSAIDs	21 (2.62%)	16 (3.08%)	5 (1.76%)	
Opioid	78 (9.71%)	51 (9.83%)	27 (9.51%)	
**Cancer types**				
Digestive organs	41 (41.41%)	26 (37.68%)	15 (50.00%)	>0.05
Respiratory and intrathoracic organs	15 (15.15%)	10 (14.49%)	5 (16.67%)	
Mesothelial	0 (0.00%)	0 (0.00%)	0 (0.00%)	
Renal and urinary tract	2 (2.02%)	2 (2.90%)	0 (0.00%)	
Thyroid and other endocrine glands	1 (1.01%)	0 (0.00%)	1 (3.33%)	
Lymphoid leukemia	16 (16.16%)	11(15.94%)	5 (16.67%)	
Malignant neoplasm of the breast	1 (1.01%)	1 (1.45%)	0 (0.00%)	
Renal cell carcinoma	0 (0.00%)	0 (0.00%)	0 (0.00%)	
Malignant neoplasm of meninges	0 (0.00%)	0 (0.00%)	0 (0.00%)	
Malignant neoplasm of spinal cord, cranial nerves, and other parts of central nervous system	0 (0.00%)	0 (0.00%)	0 (0.00%)	
Malignant neoplasm of peripheral nerves and autonomic nervous system	0 (0.00%)	0 (0.00%)	0 (0.00%)	
Malignant neoplasm brain	2 (2.02%)	1 (1.45%)	1 (3.33%)	
Malignant neoplasms	12 (12.12%)	10 (14.49%)	2 (6.67%)	
**Comorbidities**				
Cardiovascular disease	10 (22.22%)	7 (25.00%)	3 (17.65%)	>0.05
Diabetes mellitus	4 (8.89%)	2 (7.14%)	2 (11.76%)	
Hypertensive	2 (4.44%)	1 (3.57%)	1 (5.88%)	
Liver disease	3 (6.67%)	2 (7.14%)	1 (5.88%)	
Nephropathy	5 (11.11%)	3 (10.71%)	2 (11.76%)	
Pneumonia	17 (37.78%)	10 (35.71%)	7 (41.18%)	
Cerebrovascular disease	4 (8.89%)	3 (10.71%)	1 (5.88%)	

**Table 2:** Cox proportional hazard model of mortality risk by comorbidities.

Characteristic	Multivariable Cox Analysis	
	HR (95% CI)	*p*-Value
Cardiovascular disease	1.72 (0.29–10.25)	0.552
Diabetes mellitus	1.03 (0.22–4.86)	0.973
Hypertension	0.26 (0.01–4.52)	0.355
Liver diseases	1.08 (0.22–5.27)	0.928
Nephropathy	0.97 (0.26–3.68)	0.963
Cerebrovascular disease	4.34 (0.51–37.08)	0.180
Sepsis	0.66 (0.16–2.72)	0.565
Septic shock	1.27 (0.62–3.13)	0.957

**Table 3:** Risk factors associated with 168-day (6-month) mortality.

Characteristic	Univariable Analysis		Multivariate Analysis	
	HR (95% CI)	*p*-Value	HR (95% CI)	*p*-Value
**Based on Colistin use status**				
Colistin use	1.18 (0.45–3.06)	0.738	4.66 (0.74–29.45)	0.102
**Sex**				
SEX (female)	1.05 (0.29–3.73)	0.944	0.15 (0.01–1.54)	0.110
**Antibiotic susceptibility testing**				
Aminoglycoside resistance	1.66 (0.61–4.53)	0.323	54.50 (3.30–900.20)	0.005
**Concomitant Antibiotics**				
Penicillin	0.96 (0.33–2.77)	0.936	51.91 (2.83–950.60)	0.008
**Cancer Types**				
Digestive organs	0.69 (0.24–1.95)	0.483	0.02 (0.00–0.20)	0.002
Respiratory and intrathoracic organs	0.54 (0.12–2.45)	0.425	2.45 × 10^−4^ (1.49 × 10^−6^–4.02 × 10^−2^)	0.001
**Comorbidities**				
Nephropathy	0.72 (0.16–3.22)	0.667	0.021 (3.11 × 10^−4^–1.42)	0.072
Pneumonia	1.23 (0.44–3.81)	0.638	0.06 (1.94 × 10^−3^–1.85)	0.004

## Data Availability

The data presented in this study are available on request from the corresponding author due to privacy restrictions and institutional data-sharing policies.

## Supplementary Materials

The following supporting information can be downloaded at: https://www.mdpi.com/article/10.3390/medicina61081361/s1, Table S1. Baseline demographic characteristics of patients before PSM; Table S2. Standardized Mean Differences Before and After Propensity Score Matching.

## Abbreviations

ADR	Adverse Drug Reactions
AR	Antibiotic Resistance
CI	Confidence Interval
CVC	Central Venous Catheters
EMR	Electronic Medical Records
GNB	Gram-Negative Bacteria
HR	Hazard Ratio
ICD	International Classification of Disease
MDR	Multidrug Resistant
NSAIDs	Nonsteroidal Anti-Inflammatory Drugs
PD	Pharmacodynamics
PK	Pharmacokinetics
SMD	Standardized Mean Differences
TMP_SMX	Trimethoprim-Sulfamethoxazole
