# Supplementary material for: Mortality Risk of Colistin vs. Non-Colistin Use in Cancer Patients with Multidrug-Resistant Gram-Negative Bacterial Infections: Stratified by Resistance Profile and Concomitant Medications
Source: Medicina (Kaunas). 2025 Jul 28;61(8):1361. doi: 10.3390/medicina61081361 (PMC12387711; doi:10.3390/medicina61081361)
Supplement: Supplementary file 1 [file medicina-61-01361-s001.zip › medicina-3749426-supplementary.pdf]

Table S1. Baseline demographic characteristics of patients before PSM

Characteristics	No of Cases (% relative frequency) or median (IQR)		
	**Total (312)**	**Non-Colistin (276)**	**Colistin (36)**
**Age (years)**	**60.5±18.04**	**65.34 ± 12.23**	**64.64 ± 12.81**
30~39	12 (3.7%)	10 (3.47%)	2 (5.56%)
40~49	23 (7.1%)	20 (6.94%)	3 (8.33%)
50~59	60 (18.52%)	53 (18.40%)	7 (19.44%)
60~69	87 (26.85%)	79 (27.43%)	8 (22.22%)
70~79	99 (30.56%)	86 (29.86%)	13 (36.11%)
80~89	99 (9.3%)	27 (9.4%)	3 (8.3%)
**Sex**			
Men	214 (66.05%)	188 (65.28%)	26 (72.22%)
Women	98 (30.25%)	88 (30.56%)	10 (27.78%)
**Culture**			
Blood/fluid	73 (23.40%)	69 (25.00%)	4 (11.11%)
Genital/urinary	50 (16.03%)	50 (18.12%)	0 (0.00%)
Respiratory	119 (38.14%)	104 (37.68%)	15 (41.67%)
Duplicate	70 (22.44%)	53 (19.20%)	17 (47.22%)
**Gram-negative bacteria**			
Acinetobacter baumannii	54 (17.31%)	41 (14.86%)	13 (36.11%)
Klebsiella pneumoniae	108 (34.62%)	103 (37.32%)	5 (13.89%)
Pseudomonas aeruginosa	89 (28.53%)	83 (30.07%)	6 (16.67%)
⩾ 2 organisms	61 (19.55%)	49 (17.75%)	12 (33.33%)
**Death status**			
Survival	230 (70.99%)	217 (75.35%)	13 (36.11%)
Death	82 (25.31%)	59 (20.49%)	23 (63.89%)
**No. concomitantly used medications**			
1	7 (2.16%)	7 (2.43%)	0 (0.0%)
2	11 (3.40%)	11 (3.82%)	0 (0.0%)
3	4 (1.23%)	4 (1.39%)	0 (0.0%)
4	10 (3.09%)	10 (3.47%)	0 (0.0%)
≥ 5	280 (86.42%)	244 (84.72%)	36 (100.0%)
**Antibiotic resistance**			
Aminoglycoside	137 (8.39%)	110 (7.95%)	27 (10.84%)
Carbapenems	155 (9.50%)	125 (9.04%)	30 (12.05%)
Cephalosporin	219 (13.42%)	187 (13.52%)	32 (12.85%)
Cephamycins	1 (0.06%)	1 (0.07%)	0 (0.00%)
Colistin	6 (0.37%)	4 (0.29%)	2 (0.80%)
Fluoroquinolone	191 (11.70%)	158 (11.42%)	33 (13.25%)
Glycylcycline	173 (10.60%)	153 (11.06%)	20 (8.03%)
Monobactam	173 (10.60%)	144 (10.41%)	29 (11.65%)
Nitrofurantoin	3 (0.18%)	3 (0.22%)	0 (0.00%)
Penicillin	279 (17.10%)	247 (17.86%)	32 (12.85%)
Tetracycline	56 (3.43%)	46 (3.33%)	10 (4.02%)
TMP_SMX	239 (14.64%)	205 (14.82%)	34 (13.65%)
**Comedications**			
Aminoglycoside	129 (5.20%)	113 (5.32%)	16 (4.47%)
Anesthetics	214 (8.62%)	186 (8.76%)	28 (7.82%)
Antidepressants	66 (2.66%)	60 (2.82%)	6 (1.68%)
Antipsychotics	136 (5.48%)	116 (5.46%)	20 (5.59%)
Anxiolytics	22 (0.89%)	18 (0.85%)	4 (1.12%)
Benzodiazepine	236 (9.51%)	204 (9.60%)	32 (8.94%)
Buprenorphine	1 (0.04%)	1 (0.05%)	0 (0.00%)

Carbapenem	170 (6.85%)	138 (6.50%)	32 (8.94%)
Cephalosporin	279 (11.24%)	246 (11.58%)	33 (9.22%)
Fluoroquinolones	180 (7.25%)	155 (7.30%)	25 (6.98%)
Glycopeptide	179 (7.21%)	145 (6.83%)	34 (9.50%)
Hypnotics and sedatives	64 (2.58%)	53 (2.50%)	11 (3.07%)
Immunosuppressants	28 (1.13%)	22 (1.04%)	6 (1.68%)
Lincosamides	44 (1.77%)	38 (1.79%)	6 (1.68%)
Macrolide	50 (2.01%)	43 (2.02%)	7 (1.96%)
Monobactam	2 (0.08%)	1 (0.05%)	1 (0.28%)
NSAIDs	55 (2.22%)	48 (2.26%)	7 (1.96%)
Opioid	286 (11.52%)	252 (11.86%)	34 (9.50%)
Oxazolidinone	19 (0.77%)	13 (0.61%)	6 (1.68%)
Penicillin	235 (9.47%)	203 (9.56%)	32 (8.94%)
Rifamycin	16 (0.64%)	12 (0.56%)	4 (1.12%)
Tetracycline	17 (0.68%)	13 (0.61%)	4 (1.12%)
Trimethoprim-Sulfamethoxazole	35 (1.41%)	28 (1.32%)	7 (1.96%)
**Cancer types**			
Lip, oral cavity & pharynx	38 (9.62%)	18 (5.36%)	20 (33.90%)
Digestive organs	160 (40.51%)	143 (42.56%)	17 (28.81%)
Respiratory & intrathoracic organs	45 (11.39%)	37 (11.01%)	8 (13.56%)
Malignant neoplasm of bone and articular cartilage of limbs	1 (0.25%)	1 (0.30%)	0 (0.00%)
Melanoma and other malignant neoplasms of skin	2 (0.51%)	2 (0.60%)	0 (0.00%)
Mesothelial	3 (0.76%)	1 (0.30%)	2 (3.39%)
Malignant neoplasm of breast	4 (1.01%)	3 (0.89%)	1 (1.69%)
Female genital organs	13 (3.29%)	13 (3.87%)	0 (0.00%)
Male genital organs	15 (3.80%)	15 (4.46%)	0 (0.00%)
Urinary tract	22 (5.57%)	21 (6.25%)	1 (1.69%)
Malignant neoplasm of eye and adnexa	5 (1.27%)	4 (1.19%)	1 (1.69%)
Thyroid and other endocrine glands	5 (1.27%)	4 (1.19%)	1 (1.69%)
Malignant neoplasms	47 (11.90%)	45 (13.39%)	2 (3.39%)
Lymphoid leukemia	35 (8.86%)	29 (8.63%)	6 (10.17%)
**Comorbidities**			
Cardiovascular disease	40 (19.14%)	37 (19.58%)	3 (15.00%)
Diabetes mellitus	33 (15.79%)	31 (16.40%)	2 (10.00%)
Hypertensive	14 (6.70%)	13 (6.88%)	1 (5.00%)
Liver disease	36 (17.22%)	35 (18.52%)	1 (5.00%)
Nephropathy	21 (1.005%)	19 (10.05%)	2 (10.00%)
Pneumonia	40 (19.14%)	32 (16.93%)	8 (40.00%)
Cerebrovascular disease	25 (11.96%)	22 (11.64%)	3 (15.00%)

Table S2. Standardized Mean Differences (SMD) Before and After Propensity Score Matching

**Characteristic**	**Before Propensity Score matching**	**After Propensity Score matching**
	**SMD**	**SMD**
Sex (Male reference)	-0.041	-0.0011
**Antibiotic susceptibility testing**		
Aminoglycoside	0.351	-0.052
Carbapenems	0.380	-0.029
Cephalosporin	0.211	0.017
Fluoroquinolone	0.344	0.011

Monobactam	0.284	-0.034
TMP/SMX (Trimethoprim + Sulfamethoxazole)	0.202	-0.034
**Concomitant drugs**		
Benzodiazepine	0.150	-0.023
Penicillin	0.153	0.029
Carbapenem	0.389	0.034
Glycopeptide/Tetracyclines	0.419	0.017
Oxazolidinone	0.120	-0.046
**Cancer Types**		
Digestive organs	-0.046	0.017
Respiratory and intrathoracic organs	0.088	0.011
Mesothelial	0.052	0.000
Malignant neoplasm of breast	0.017	-0.017
Female genital organs	-0.047	0.000
Male genital organs	-0.054	0.000
Urinary tract	-0.048	-0.023
**Comorbidities**		
Liver disease	-0.099	0.011
Cardiovascular disease	-0.051	0.006
Diabetes mellitus	-0.057	0.040
Hypertensive	-0.019	0.023
Nephropathy	-0.010	0.006
Pneumonia	0.106	0.034
Cerebrovascular disease	0.004	-0.006
